# Corticospinal Tract Tracing in the Marmoset with a Clinical Whole-Body 3T Scanner Using Manganese-Enhanced MRI

**DOI:** 10.1371/journal.pone.0138308

**Published:** 2015-09-23

**Authors:** Boris Demain, Carole Davoust, Benjamin Plas, Faye Bolan, Kader Boulanouar, Luc Renaud, Robert Darmana, Laurence Vaysse, Christophe Vieu, Isabelle Loubinoux

**Affiliations:** 1 Inserm, Imagerie cérébrale et handicaps neurologiques, UMR 825, F-31024, Toulouse, France; 2 Université de Toulouse, UPS, Imagerie cérébrale et handicaps neurologiques, UMR 825, CHU Purpan, Place du Dr Baylac, F-31059, Toulouse, Cedex 9, France; 3 CNRS-LAAS, 7 avenue du colonel Roche, F-31077, Toulouse, France; 4 Pôle Neurosciences, Centre Hospitalier Universitaire de Toulouse, Toulouse, France; 5 CNRS, Centre de Recherche Cerveau & Cognition, UMR 5549, F-31024, Toulouse, France; Uniformed Services University, UNITED STATES

## Abstract

Manganese-enhanced MRI (MEMRI) has been described as a powerful tool to depict the architecture of neuronal circuits. In this study we investigated the potential use of in vivo MRI detection of manganese for tracing neuronal projections from the primary motor cortex (M1) in healthy marmosets (Callithrix Jacchus). We determined the optimal dose of manganese chloride (MnCl_2_) among 800, 400, 40 and 8nmol that led to manganese-induced hyperintensity furthest from the injection site, as specific to the corticospinal tract as possible, and that would not induce motor deficit. A commonly available 3T human clinical MRI scanner and human knee coil were used to follow hyperintensity in the corticospinal tract 24h after injection. A statistical parametric map of seven marmosets injected with the chosen dose, 8 nmol, showed the corticospinal tract and M1 connectivity with the basal ganglia, substantia nigra and thalamus. Safety was determined for the lowest dose that did not induce dexterity and grip strength deficit, and no behavioral effects could be seen in marmosets who received multiple injections of manganese one month apart. In conclusion, our study shows for the first time in marmosets, a reliable and reproducible way to perform longitudinal ME-MRI experiments to observe the integrity of the marmoset corticospinal tract on a clinical 3T MRI scanner.

## Introduction

The corticospinal tract (CST) or pyramidal tract is the motor output from the brain to the spinal cord. The CST is involved in two major components of motricity: strength and dexterity [1]. Nerve fibers in the corticospinal tract originate from pyramidal cells in layer V of the primary motor cortex (about 30%), the supplementary motor area and the premotor cortex (together also about 30%), and also the somatosensory cortex, parietal lobe, and cingulate gyrus. The CST is frequently affected in stroke and its integrity has been correlated to functional motor recovery [2]. Only 5–20% of patients with a brain injury will entirely recover normal upper limb motor function following physical therapy. Usually limb strength, and more markedly dexterity remain impaired. Non-invasive imaging techniques have been proposed to assess the integrity of the CST in longitudinal studies. They are based on the Diffusion-Tensor MRI and the fiber tracking uses the anisotropic property of water diffusion in the white-matter (WM). To reconstruct the tracks, the quantity and directionality of the anisotropic diffusion of the WM are measured through the fractional anisotropy or relative anisotropy and displayed as maps. However, direct visualization of the CST is not possible on these images and a priori knowledge is needed [3]. Besides these non-invasive MRI techniques, neuroanatomical tracing studies require fixed tissue, and therefore sacrifice of animals for data analysis. Manganese is a MRI contrast agent that traces neuronal pathways. Thus, manganese-enhanced magnetic resonance imaging (ME-MRI) has been introduced as an in vivo method for visualization of neuronal tracts [4]. The manganese ion (Mn^2+^), acting as a Ca^2+^ analogue [5,6], is taken up into the intracellular space by L-type voltage-gated calcium channels and activates glutamate receptors (e.g., N-methyl-D-aspartate receptors) [6–8][9]. Mn^2+^ can then diffuse throughout the cytoplasm and is transported down axons by microtubules [10]. Manganese acts as a contrast agent on T1-weighted MR images. The fact that Mn^2+^ moves in an anterograde direction along the appropriate neuronal pathway and can cross synapses has led to the possibility of using ME-MRI to detect functional neural circuits [4,11–13].

Since the technique was introduced, many ME-MRI tract tracing studies in living animals have been performed successfully and have revealed specific neuronal connections such as in the olfactory tract [4,14], visual optic tract [4,15–18], corticospinal tract [19–26], hindlimb and face somatosensory tracts [19,22], basal ganglia tracts [13], auditory tract [27], and song control pathway in songbirds [28,29][9].

Since the marmoset is more dexterous than the rodent, it is a relevant non-human primate model to explore the CST and its integrity. In previous reports, manganese has been injected into the eye of marmosets [18] or in the tail vein [30] and was shown to be safe. It is yet to be injected into the primary motor cortex to label the CST. What’s more, safety of intracerebral manganese injection has rarely been tested on behavior. In our study, strength and dexterity assessment was performed to test the functionality of the corticospinal tract post- injection.

The first aim of the study was to assess the feasibility of ME-MRI injected in the brain primary motor cortex (M1) marmoset in a clinical MRI scanner fitted with a human knee coil. The second aim was to determine the optimal dose of manganese for a specific tracing of the CST. Finally, the third aim was to explore the behavioral consequences of a single manganese injection on specific tasks centered on the corticospinal tract functions, force and dexterity. Since multiple injections were performed on each animal, the safety of manganese injections could also be assessed.

## Materials and Methods

### 2.1 Ethics Statement

The entire study was approved by the « Direction départementale de la Protection des Populations de la Haute-Garonne » and « Comité d’éthique pour l’expérimentation animale Midi-Pyrénées ». This protocol obtained authorization n°31125507.

### 2.2 Animals

Experiments were performed on 7 common marmosets (Callithrix Jaccus) wich coming from our own colony, 3 males and 4 females aged 4.5 ± 1.1 years (mean ± standard deviation), and weighing 349g ± 27g. Marmosets were housed alone (for their security) or two by cage, there were ten cages in the same room and marmosets could see and hear between them. They had access to tunnel and nest like enrichment. They lived with a 12h light/dark cycle following European norms accredited by « Direction Départementale de la Protection des populations » with authorization n°A3155501.

None of the animals were sacrificed at the end of the experiments and therefore they can integrate with other protocols.

### 2.3 Surgery and hydrated manganese chloride injection (MnCl_2_)

Food was withdrawn 12 hours before surgery. A 20 μg/kg solution of glycopyrrolate (Robinul-V®, Vetoquinol, Lure, France) was administered with an intramuscular (IM) injection, 10 min before anesthesia. Light anesthesia was induced with an IM injection of Alfaxalone (7 mg/kg, Alfaxan®, Worcestershire, UK). All marmosets received premedication by IM injections of Oxytetracycline (20 μg/kg, Terramycine longue action®, Pfizer, Paris, France), Buprenorphine (25 μg/kg, Vetergesic®, Reckitt Benckiser Healthcare, Danson, UK), Methylprednisolone (5 mg/kg, Solumedrol®). First, the head was shaved with a surgical shearer followed by shaving of the scalp using a razor. Blood oxygen saturation, respiratory rate and heart rate were continuously monitored throughout the entire surgical procedure (Starr Life Science, Oakmont, PA, USA). Rectal temperature was maintained around 37.5 ± 0.5°C by a heating pad. Animals were positioned on a rat stereotaxic frame with ear bars adapted for marmoset and were anesthetized with 2% isoflurane in 80% O_2_/20% air. Oxygen saturation was maintained over 95%. Thorough sterilization of the skin was achieved with three washes with betadine/alcohol 70%. A 2 cm scalp incision was made, followed by drilling of the cranium at the coordinates for forelimb primary motor cortex and thus the site of injection: 6 mm anterior and 4.5 mm lateral relative to Bregma according to the Stereotaxic Atlas of the Marmoset Brain [31]. The needle was inserted 2.5 mm deep from the brain surface.

Solutions of MnCl_2_ (Sigma Aldrich, France), 5M (n = 1), 2.5M (n = 2), 0.5M (n = 2), 0.25M (n = 1), 0.05M (n = 7) were injected with consistent volume of 0.16μl so that 800, 400, 80, 40, and 8 nmol were injected respectively. The solution was injected slowly using a Hamilton microsyringe 701N fitted with a needle with an external diameter of 0,22mm (Phymep, Paris, France) at a rate of 0.02 μL/min. The needle was removed 5 min after complete injection to avoid reflux. The hole in the skull was sealed using dental cement filling (Paladur, Heraeus, Germany). Finally, both the galea and scalp were sutured with 5/0 and 3/0 thread (Vicryl), respectively. Isoflurane was stopped and oxygen was maintained until signs of wakefulness appeared. Marmosets were returned to their cages with access to food and water ad libitum. Three marmosets had multiple injections of manganese (up to four) administered at least one month apart.

### 2.4 *In vivo* MRI

Twenty four hours following MnCl_2_ injection, marmosets were imaged on a 3T Achieva (Phillips) MRI scanner fitted with a human knee coil. For each experiment, two animals were anesthetized by IM injection of Alfaxalone 18 mg/kg and positioned head to head, supine in a home-made cradle. A high resolution 3D T1-weighted magnetization prepared gradient-echo (MPRAGE) sequence was performed on the marmosets (TR/TE = 11/4.9ms, TI = 810 ms, FOV = 135 x 95 x 45 mm, matrix = 336 x 136 x 112, voxel size 0.4 x 0.4 x 0.7, reconstructed 0.4 x 0.4 x 0.4 mm, flip angle = 8°, NSA = 4, 23 min). Images of each marmoset were individualized and reconstructed aligned to the bicommissural plane.

### 2.5 Image analysis and statistics

**Fig 1:**
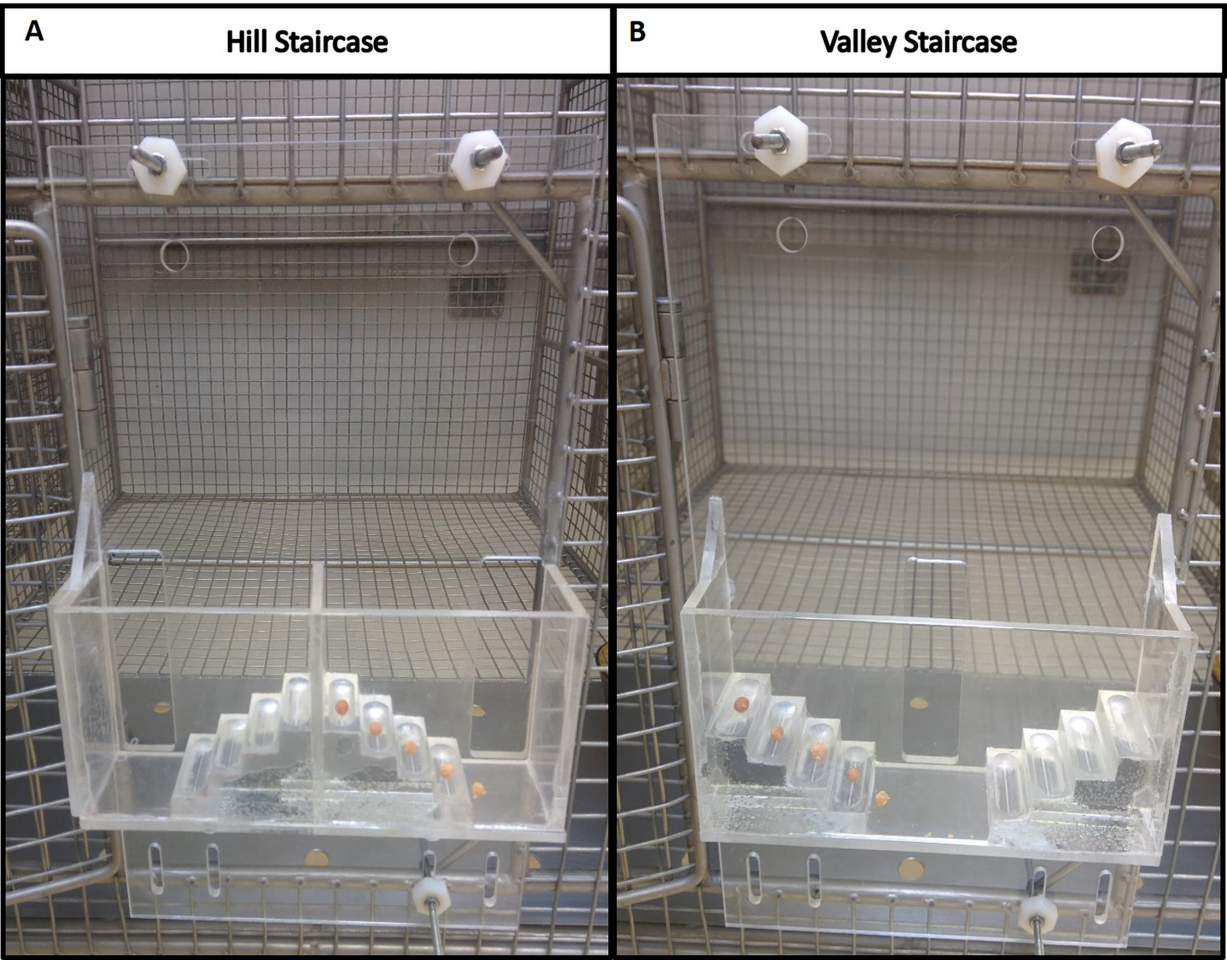
Four MnCl_2_ doses shown 24h post injection in marmoset brain. (A) Slices at the injection site (+6mm from bregma), and (B) slices 2 mm posterior to the injection site. Top: Raw images. Bottom: ROI of MnCl_2_ hyperintensity automatically thresholded at 195 on the grey scale (256 levels). To the right of the figure, corresponding slices of the Atlas of Yuasa et al, 2010. The following structures are hyperintense: primary motor cortex M1 (Brodmann area 4), the primary sensory cortex (3a), the cingulum (23–24), the premotor cortex (6c,6d), the parietal cortex (5), corpus callosum, corona radiata, caudate (Cd), putamen (Pu), internal (IGP) and external (EGP) globus pallidus, thalamic nuclei (VL: ventral lateral, RT: reticular), the internal capsule (ic). Note that MnCl_2_ follows the corpus callosum to the contralateral hemisphere most significantly with the highest doses.

The MRI data were preprocessed using Image J software v1.45s (National Institutes of Health, USA) allowing us to extract the brain from the images by manual drawing of a ROI. Before drawing, the images were scaled to have a maximum value of 255. A threshold of 195/255 was applied to each image to select hyperintense ROIs (in green on Fig 1).

After dose selection, the images obtained at 8 nmol of MnCl_2_ were statistically analyzed using SPM 8 software (Wellcome Department of Imaging Neuroscience www.fil.ion.ucl.ac.uk/spm) implemented in Matlab (MathWorks, Natick, MI, USA). All images were normalized to the Japanese template [32] and smoothed at 0.8 mm in the three directions. For comparison of the manganese injected hemisphere to the contralateral hemisphere, the seven images were compared pixel to pixel to their flipped counterpart in a paired t-test. A Statistical MnCl_2_ map was obtained and a thresholded was set at p<0.005 uncorrected, extent threshold n = 150. The origin was set according to the atlas of Hikishima at the center of the interaural line.

### 2.6 Behavioral testing

Animals were acclimated and trained five times a week with the behavioral tests for 2 months prior to the MRI exam. Once trained, the baseline value was determined by the mean of the three last measured values. Food and water deprivation was not required before testing. Testing was performed early in the afternoon. The marmosets were fed with their normal daily diet after behavioral testing. Behavioral testing to detect potential motor deficiencies was then performed four and six days post MnCl_2_ injection.

#### 2.6.1 Hill and Valley Staircases

**Fig 2:**
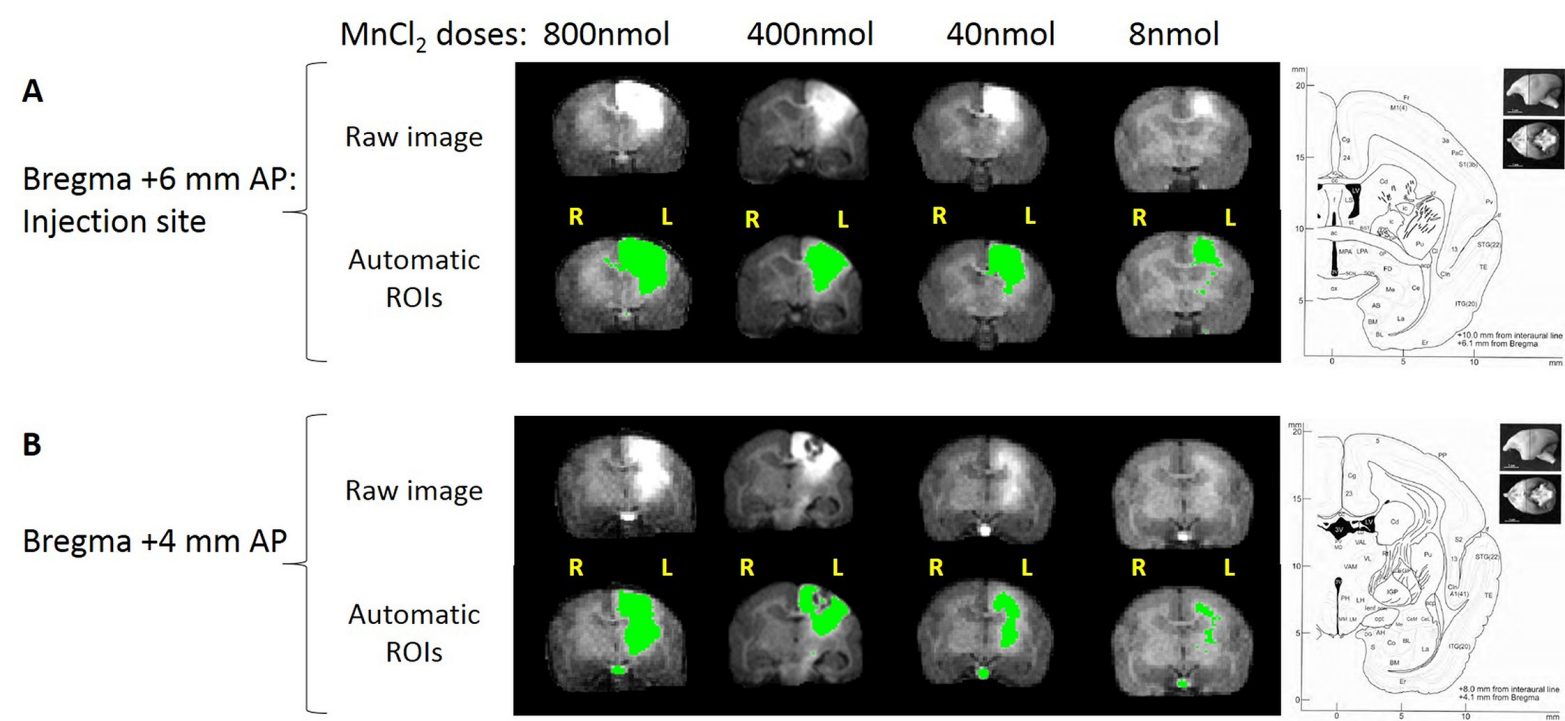
Marmoset staircase. Picture of Hill (A) and Valley (B) staircases attached to the front of the marmoset cage. Steps are baited for left forelimb testing.

These tasks measure the marmoset’s ability to reach and grasp a food reward. The rewards were gingerbread pellets placed on five steps of two staircases located behind a Plexiglas screen attached to the front of the cage (Fig 2). In the Hill version, entry is via a lateral slot (situated at the both sides of the staircase) so that the animal uses its right forelimb to retrieve the reward situated on the right stair (left forelimb for the left stair). In the Valley version, there is a central slot and the animal uses its left forelimb to reach the reward situated on the right stair, and vice versa. The apparatus were modified from the Marshall Staircase [33]. Each plexiglas step was drilled 3mm deep along its 3 cm length to create a well and make pellet retrieval harder. As in Marshall’ Staircase test, a score was assigned to each step: 1 for the lowest to 5 for the upper step so that the maximal mark for each arm was 15 points. Unlike Marshall’s study, points were withdrawn if a pellet fell to another step (for example if the pellet on step 5 fell to step 3, the marmoset loses 2 points). The two versions of the test, Hill and Valley tasks, were performed on each arm and performances were timed.

#### 2.6.2 Dynamometric pull test

This task measures the strength and grasping ability of the marmoset. A bar is linked to a dynamometer. The marmoset must pass the arm through the bars of the cage to grasp the handle. If it grasped the bar correctly, it was given a gingerbread pellet. Timing was recorded.

## Results

### 3.1 Feasibility on a clinical scanner

The images were performed on a 3T Achieva (Phillips) MRI scanner equipped with standard human knee coil. Two animal heads were scanned together to improve the signal to noise ratio. The white matter/grey matter contrast was sufficient to visualize key structures such as the anterior commissure, the corpus callosum, external and internal capsules. The hyperintensity following manganese injection was clearly visible 24h after.

### 3.2 Dose selection

A high dose, 800 nmol, was injected first in order to detect the maximal extent of manganese induced hyperintensity from the injection site. The manganese spread out from the injection site and outside the primary motor cortex to the cingulum, premotor and sensory cortices, and projected to many subcortical areas (Fig 1, see atlas views) whereas hyperintensity could not be detected further than the pyramids of the medulla. Thus, lower doses were tested, 400, 40 and 8 nmol. A reliable signal could be seen in the internal capsule at the lowest dose of 8 nmol. This dose proved to be the most effective for specifically tracing the corticospinal tract, which was particularly visible on the slice 2 mm posterior to the injection site (Fig 1B). An automatic threshold was applied to highlight the manganese hyperintense tracts on the images. An artifact identified as a zone of low signal intensity could be seen sometimes around the injection site due to a hyper-concentration of manganese that could be explained by T2* intravoxel dephasing from the magnetic susceptibility [22] (Fig 1B, dose 400 nmol). It was noted that MnCl_2_ followed the corpus callosum to the contralateral hemisphere with the highest doses. However, the tracing towards the contralateral hemisphere was low at the smallest 8 nmol dose. The 8 nmol concentration was chosen and then seven marmosets were injected and scanned with this dose.

### 3.3 Manganese tracts

**Fig 3:**
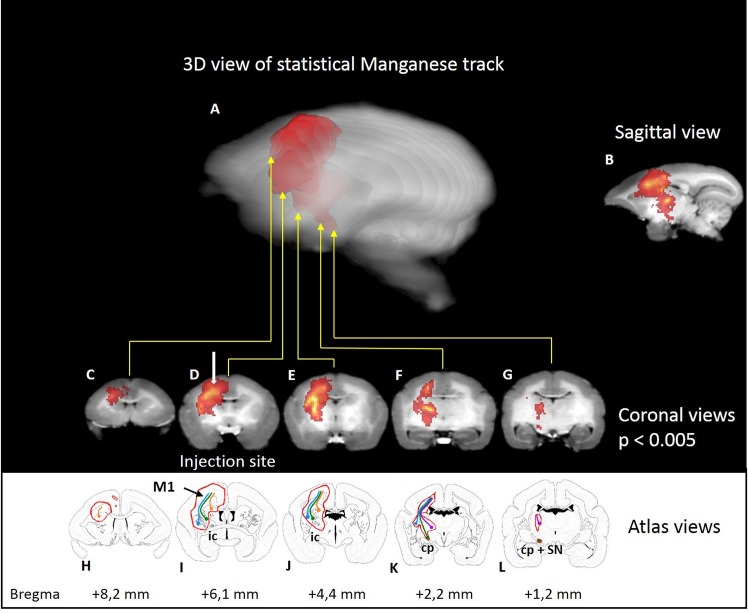
Statistical parametric maps of manganese in seven marmosets. Statistical parametric maps of manganese after injection (blue point) in the primary motor cortex (M1) of seven marmosets (p<0.005 uncorrected). A: Semitransparent three-dimensional MnCl_2_ maps on the single brain template [32]. Animals were imaged 24h after 8nmol MnCl_2_ injection. MnCl_2_ induced hyper intensity on brain T1-weighted images. C-G: coronal views. H-L: Corresponding marmoset brain atlas in coronal view. The following structures are marked: M1 primary motor cortex, Cd: Caudate nucleus, Pu: putamen, GP: globus pallidus, ic: internal capsule, cp: cerebral peduncule and SN: subtantia nigra, the cingulum, the premotor cortex (Brodmann area 6c,d), the parietal cortex (Brodmann area 5), corpus callosum, corona radiata, thalamic nuclei (VL: ventral lateral thalamic nucleus, VPL: ventral posterolateral thalamic nucleus, VPM: ventral posteromedial thalamic nucleus, CM: central medial thalamic nucleus, RT: reticular thalamic nucleus). Colored lines indicate the cortico-caudate tract (orange), the corticospinal tract (green), the cortico-putaminal tract (blue), and the cortico-thalamic tract (purple).

**Fig 4:**
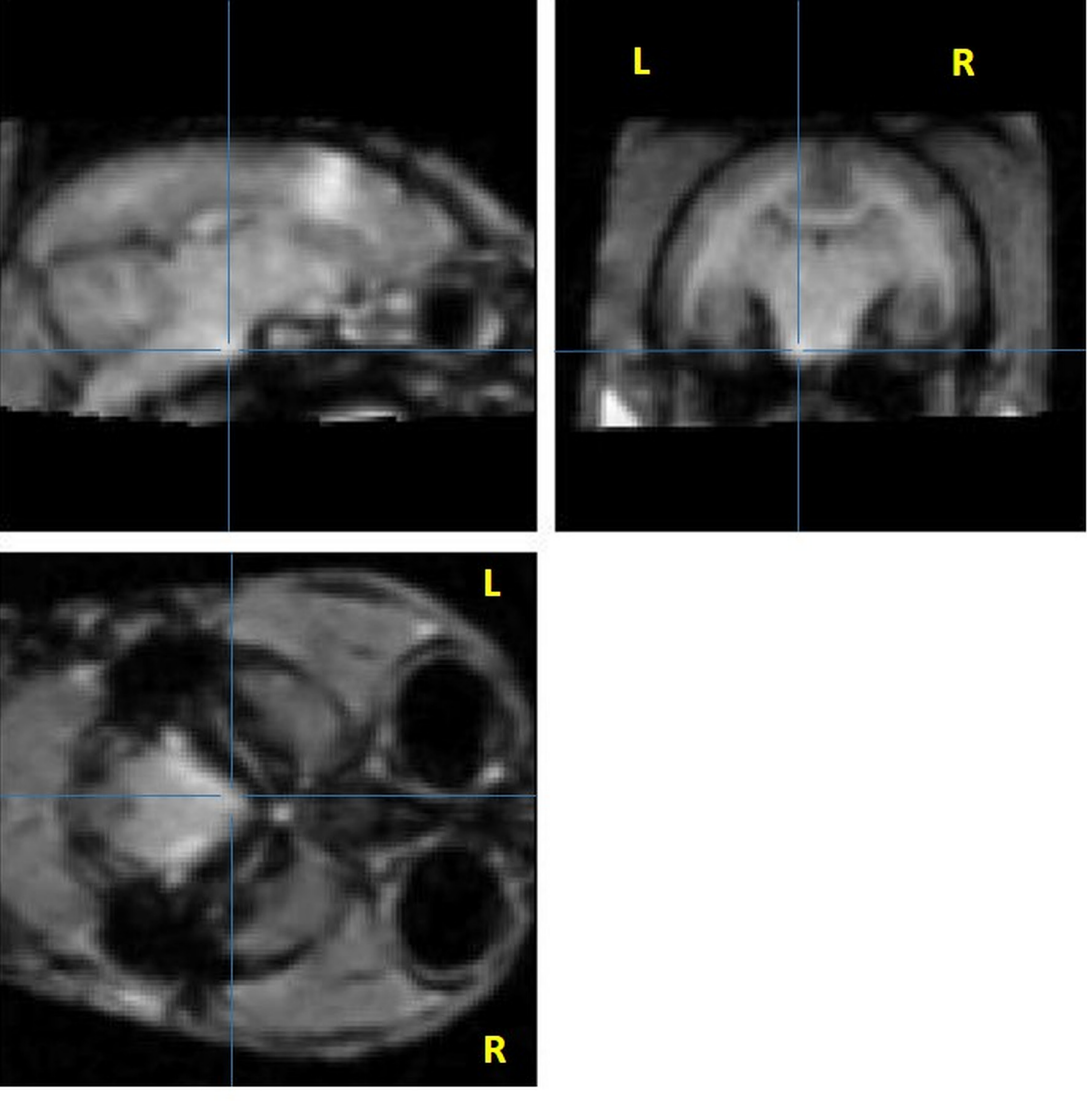
Single marmoset’s raw image showing manganese-induced hyperintensity in the pyramids. Sagittal, coronal, and axial slices showing manganese-induced hyperintensity in the pyramids. Note the hyperintensity compared to the contralateral side. Raw images of the marmoset with MnCl2 signal reaching the pyramid with an 8 nmol injection. Three views at coordinates 1,25mm lateral; -0.2 mm AP from bregma; +3 mm dorsoventral from the interaural line. Lines show MnCl2 signal in the pyramid at the brainstem level.

To detect the tracks, we tried applying an automatic threshold (see above) which proved to be unsatisfactory for correct detection of hyperintensity as it did not take into account the differences in contrast of grey matter and white matter, or interindividual differences. Consequently, we then processed the images from a group of 7 marmosets using SPM software. The injected hemisphere was compared to the contralateral hemisphere by flipping the brain images and calculating the statistical difference. Significant hyperintensities were evident in the primary sensorimotor cortex and the corticospinal tract: the subcortical white matter, the corona radiata, the internal capsule, and the cerebral peduncle. Adjacent structures were also partly labelled: the cingulum, the premotor cortex (Brodmann areas 6c, 6d), the parietal cortex (Brodmann area 5), corpus callosum, and from either side of the internal capsule, the caudate-putamen, globus pallidus, thalamic nuclei (VL: ventral lateral, VPL: ventral posterolateral, VPM: ventral posteromedial, CM: central medial, RT: reticular), and the substantia nigra (Fig 3). No significant voxels were detected in the pyramids at a p < 0.005 (uncorrected) threshold. However, some individuals displayed some hyperintensity in the pyramid ipsilateral to the injection (Fig 4), this did not reach significance at the group level. The furthest extent of manganese induced hyperintensity was 15.3mm in one marmoset, spanning from M1 to the pyramids.

Manganese labeled not only the M1 but secondary cortices too: the cingulum, the premotor cortex, the primary sensory cortex and the parietal cortex. Accordingly, subcortical structures were also hyperintense such as the caudate nucleus, the putamen and the thalamus through these connections. These tracts are identified by colored lines on Fig 3, the cortico-caudate tract (orange), the corticospinal tract (green), the cortico-putaminal tract (blue), and the cortico-thalamic tract (purple). Finally, manganese could also pass through synapses and the external globus pallidus and thalamus are labelled through the well-known cortico-putamino-pallido-thalamic loop, the substantia nigra through the striatum connection.

### 3.4 Behavioral correlates

**Fig 5:**
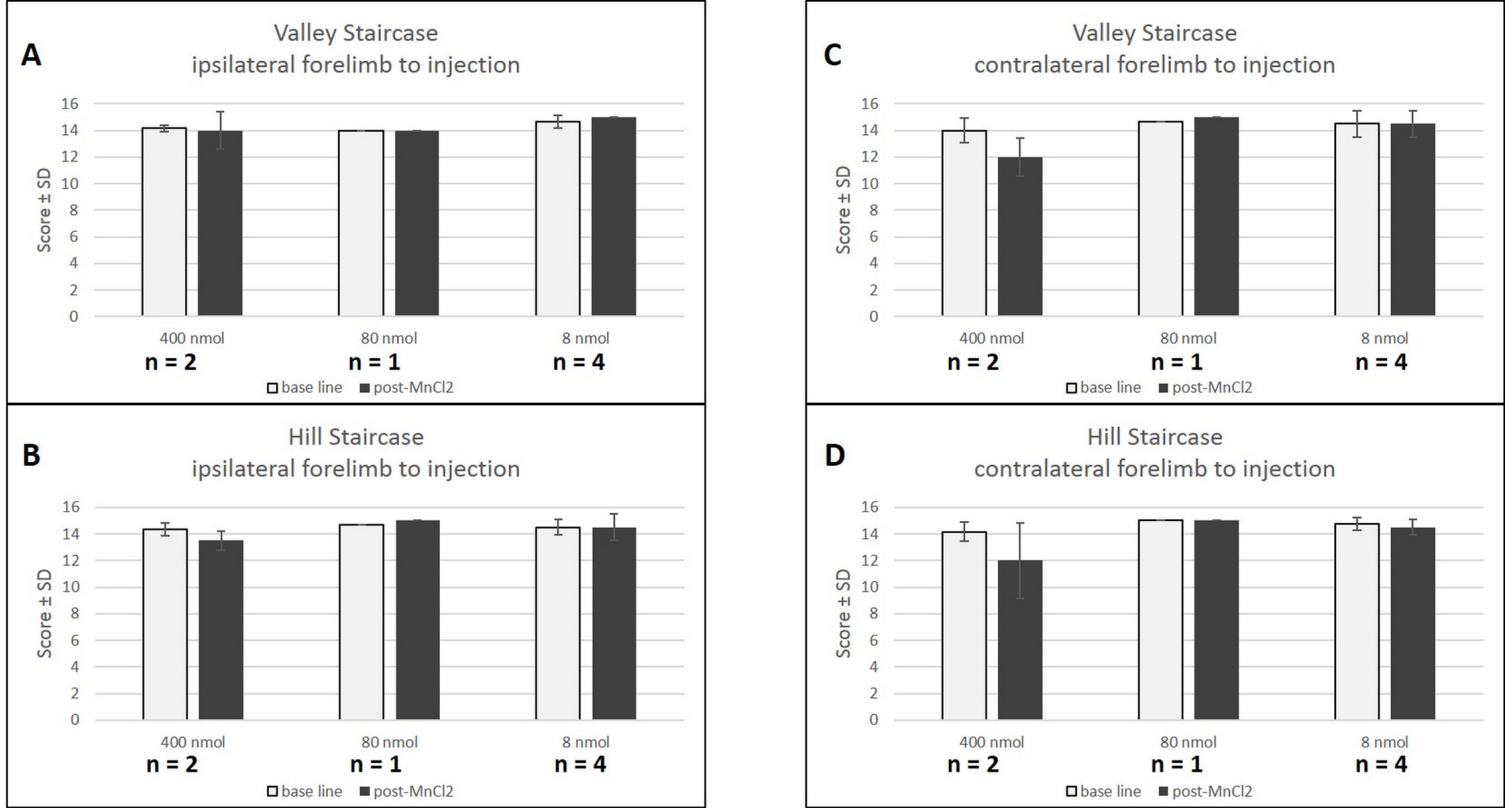
Behavioral effect of MnCl_2_ injection. Scores (number of pellets ± SD) at the Valley (A,C) and Hill (B,D) staircase before (white) and after (black) contralateral (C,D) and ipsilateral (A,B) MnCl_2_ injection. No behavioral deficits are observed after low concentrations (80 and 8 nmol), four days after injection. However the high concentration (400 nmol) caused a decrease in valley and hill scores only in the contralateral forelimb. Baseline scores are represented in white while 4 days post-injection of MnCl_2_ scores are represented in black.

Dexterity and grip strength were measured before, four days, and six days post injection of manganese. The animal injected with 800 nmol of Mn was isolated following a paw injury sustained during a fight with another marmoset and therefore could not be tested. With both versions of the staircase (valley and hill), the high concentration (400 nmol) induced a mean decrease of performance of 2 points in the forelimb contralateral to the injection (Fig 5C and 5D) four days post- injection in each marmoset while no deficit was seen in the ipsilateral forelimb. In contrast, no behavioral deficit was observed after low concentrations (80 and 8 nmol), four days post- injection, for either forelimb. The deficits after the high dose were transient and motor behavior was normal six days after injection (scores at 15 for the two marmosets). With the dynamometric pull test, there was no grip deficit at high concentration (400 nmol, n = 2). Thus, this test seems less sensitive than the staircase test.

Three marmosets were given multiple injections of manganese (up to four injections, with a maximum sum of 888 nmol (400, 400, 80 and 8 nmol in one animal administered at least one month apart). No adverse behavioral effects could be seen acutely or in the long-term in these animals.

## Discussion

In this study, we demonstrate that the 3T Achieva (Phillips) MRI scanner equipped with a standard human knee coil is sufficient to get good detection of the contrast agent MnCl_2_ on T1-weighted images for rapid, in vivo and longitudinal tracing of major cortico-cortical and cortico-subcortical projections in the marmoset after an injection in the primary motor cortex. Until now, no study has explored such tracing after focal injection of manganese using a common clinical 3T MRI scanner and human coil in marmosets. Leergard et al. and Dyrby et al. used a small homemade coil on rats and minipigs respectively [21,22]. However, human 3T MRI equipment does not allow us to specifically study layer connectivity as done by Tucciarone et al. [34]. To improve the signal to noise ratio, we performed MRI examination simultaneously on two marmosets aligned head to head. However, scanning one animal at a time was also feasible and yielded roughly the same image quality. Over all, with the low 8 nmol dose, no major inter-individual difference in the tracing extent was visible, all injections successfully led to a hyperintense CST, and inter-individual variability was taken into account in the statistical analysis.

The common marmoset is a promising model in biomedical and neuroscience research [35]. Few ME-MRI studies have been carried out in non-human primates. Past studies of manganese in marmosets include injection into the eye to follow the visual tracts [18] and systemic injection to explore toxicity in brain areas [36,37]. However, to our knowledge, no focal injection of the motor cortex has been done to observe connectivity as previously done in rats [19,26,38,22,25].

Quantitative evaluation of contrast enhancement showed that 24h post MnCl_2_ injection would be the best time-point for manganese tracing using MRI in living rats, with the best signal to noise ratio [19]. However, Daoust et al. [39] proposed an earlier optimal time-point for more selective labelling, 2 hours after MnCl_2_ injection. At this point, the ME-MRI signal was hyperintense at the injection site and in some structures of the ipsilateral hemisphere but not in the contralateral hemisphere, while 6–10 hours post injection, the ME-MRI signal extended to all ipsi- and contralateral structures that were associated with the somatosensory pathways [39]. Twenty-four hours after injection of manganese compared to 10h, the ME-MRI signal was more diffuse but corticothalamic structures could still be clearly depicted [39]. It must be noted that manganese tracing is transynaptic and can also trace retrogradely [9]. Soria et al., [40] showed that 7 days after MnCl_2_ injection, manganese-induced hyperintensity was no longer detectable except at some points surrounding the injection site, and showed a complete loss of manganese-induced contrast 15 days after injection. Due to the marmoset brain being approximately four times bigger than that of the rat, the regulatory open-hours of the primate housing and our interest in labelling a large sensorimotor network arising from M1 involving transynaptic passages, we chose to perform scans 24h after MnCl_2_ injection.

We have determined the optimal dose of manganese to ensure labelling as specific as possible, which travelled furthest along the CST. Bilgen et al., demonstrated labeling as far as the spinal cord with electrical cortical stimulation but their images suggest a loss of cortical specificity when compared to none stimulation [20]. The 8 nmol dose was chosen and 7 marmosets were injected. Without electrical stimulation, labelling of the cerebral peduncle was statistically detectable with a small group of animals, and enhanced signal of the pyramid in the brainstem was sometimes directly visible on raw individual images. Since propagation of manganese in descending corticofugal pathways has been measured at around 1.4–6.1 mm/h [4,13,22,28,41], a scanning acquisition 24h post injection was highly sufficient to reach the spinal cord. However, not enough manganese reached the spinal cord to be visible on T1-weighted images. CST explorations by ME-MRI need to tackle the problem of specificity of cerebral manganese labelling as, high doses result in loss of specificity of the tract. According to Allegrini et al., Daoust et al. and Tucciarone et al., we also find labelling of the interhemispheric pathway in the corpus callosum [19,34,39]. However, the contralateral hemisphere was reached only with the highest dose (800nmol). Since we aimed to focus on a primary motor cortex labelling to see its specific CST, high doses that spread wide to the cingulum, premotor cortices (800, 400, 80 nmol), and primary sensory (S1) and parietal cortices (800, 400 nmol) were not specific enough (Fig 1). However, limiting the labelling to M1 only could not been achieved. Limited but significant labelling of premotor and parietal cortices was observed. Thus, the present study explores the corticospinal tract also originating from these neighboring areas.

The statistical parametric maps of manganese distribution in seven marmosets (p<0.005 uncorrected) show either a spread of the manganese in the cortices, or a cortico-cortical connectivity with the cingulum, the premotor cortex (Brodmann area 6c, 6d), and the parietal cortex (5). Furthermore, cortico-subcortical connectivity was observed since specific tracts could be seen in subcortical white matter, corona radiata, internal capsule and cerebral peduncle, leading to subcortical structures: caudate putamen, globus pallidus, thalamic nuclei and subtantia nigra (Fig 3). The labelling of this sensorimotor network is in agreement with the literature based on rats [19,22,25,26,38].

As MnCl_2_ is taken up by Ca^2+^ channels of activated neurons [6,8], the more CST activity the better the signal will be. Since ME-MRI aims to show integrity of the CST, it is very important to avoid anatomical lesions or motor deficits, whilst maintaining a good detection threshold. Systemic injection of 4 x 30 mg/kg MnCl_2_ induces an accumulation of the compound in the basal ganglia [36]. This accumulation can lead to manganism, which displays clinical signs similar to those of Parkinson’s. We did not see any suchlike symptoms even with focal injection at high doses (800 nmol, less than 0.5mg/kg). Canals et al. (2008) define a threshold in rats of 16 nmol for manganese toxicity at the injection site regarding neuronal cell death, and of 8 nmol for astrogliosis in an approximate tissue volume of 8–11 μL. Simmons et al. demonstrated that a high dose of manganese, 400 nmol injected in rhesus monkey brain, did not cause: cell death at the injection site, damage to downstream targets of manganese transport, changes in neuronal responsiveness and more importantly no behavioral deficits in a test of visual discrimination and a cognitive task [42]. With the same dose but in a smaller brain, we observed behavioral deficits in marmosets, which were not observed with the 80 nmol dose. Thus, the threshold for behavioral toxicity in marmoset was between 80 and 400 nmol according to the sensitivity of the staircase tests. Since we injected 8 nmol in the marmosets, it is very unlikely that this dose presented cell toxicity and our low doses (< 80 nmol) of manganese were found to be safe regarding strength and dexterity tasks. Three of the marmosets had multiple injections of manganese (up to four injections), administered one month apart with a maximum cumulated dose of 888 nmol. This was a higher dose than that of Soria et al. who gave 3 x 60 nmol at 15 days intervals. Multiple injections of low dose (8 nmol) manganese seemed safe and did not lead to motor deficits. This opens an avenue for the use of ME-MRI in longitudinal studies to explore integrity and recovery of the CST following stroke and associated therapies in small monkeys. Moreover fMRI and ME-MRI studies could potentially be performed simultaneously in the same animals to explore compensatory functional areas [40].

## Conclusion

In summary we evidence here for the first time, tracing of the sensorimotor network in the marmoset brain using manganese chloride performed on a clinical 3T MRI scanner equipped with a standard human knee coil. This is commonly available clinical equipment and therefore our protocol could benefit a wide community of researchers. As we monitored the motor behavior, our study shows a reliable and reproducible way to perform longitudinal ME-MRI experiments to observe the integrity of the marmoset corticospinal tract. This could be used to explore how the CST modulates connectivity associated with learning, plasticity and pathological states such as stroke. Effects of therapeutic treatments that protect or stimulate axon growth could also be analyzed with this method.
